# N-linked glycosylation of the West Nile virus envelope protein is not a requisite for avian virulence or vector competence

**DOI:** 10.1371/journal.pntd.0007473

**Published:** 2019-07-15

**Authors:** Payal D. Maharaj, Stanley A. Langevin, Bethany G. Bolling, Christy C. Andrade, Xavier A. Engle, Wanichaya N. Ramey, Angela Bosco-Lauth, Richard A. Bowen, Todd A. Sanders, Claire Y.-H. Huang, William K. Reisen, Aaron C. Brault

**Affiliations:** 1 Division of Vector-Borne Infectious Diseases, Centers for Disease Control and Prevention, Fort Collins, CO, United States of America; 2 Center for Vector-borne Disease Research and Department of Pathology, Microbiology and Immunology, School of Veterinary Medicine, University of California, Davis, Davis, CA, United States of America; 3 Department of Biomedical Sciences, Colorado State University, Fort Collins, CO, United States of America; 4 U.S. Fish and Wildlife Service, Vancouver, WA, United States of America; University of Florida, UNITED STATES

## Abstract

The N-linked glycosylation motif at amino acid position 154–156 of the envelope (E) protein of West Nile virus (WNV) is linked to enhanced murine neuroinvasiveness, avian pathogenicity and vector competence. Naturally occurring isolates with altered E protein glycosylation patterns have been observed in WNV isolates; however, the specific effects of these polymorphisms on avian host pathogenesis and vector competence have not been investigated before. In the present study, amino acid polymorphisms, NYT, NYP, NYF, SYP, SYS, KYS and deletion (A’DEL), were reverse engineered into a parental WNV (NYS) cDNA infectious clone to generate WNV glycosylation mutant viruses. These WNV glycosylation mutant viruses were characterized for *in vitro* growth, pH-sensitivity, temperature-sensitivity and host competence in American crows (AMCR), house sparrows (HOSP) and *Culex quinquefasciatus*. The NYS and NYT glycosylated viruses showed higher viral replication, and lower pH and temperature sensitivity than NYP, NYF, SYP, SYS, KYS and A’DEL viruses *in vitro*. Interestingly, *in vivo* results demonstrated asymmetric effects in avian and mosquito competence that were independent of the E-protein glycosylation status. In AMCRs and HOSPs, all viruses showed comparable viremias with the exception of NYP and KYS viruses that showed attenuated phenotypes. Only NYP showed reduced vector competence in both *Cx*. *quinquefasciatus* and *Cx*. *tarsalis*. Glycosylated NYT exhibited similar avian virulence properties as NYS, but resulted in higher mosquito oral infectivity than glycosylated NYS and nonglycosylated, NYP, NYF, SYP and KYS mutants. These data demonstrated that amino acid polymorphisms at E154/156 dictate differential avian host and vector competence phenotypes independent of E-protein glycosylation status.

## Author summary

West Nile virus (WNV) has been responsible for the largest human encephalitis epidemics in the continental United States. Avians and *Culex* mosquitoes are the primary hosts for WNV natural transmission cycles. The envelope (E) protein for WNV contains a variable N-linked glycosylation motif which influences avian replication, mosquito infectivity and vector competence. WNV isolates with variable E protein glycosylation motifs that have been historically associated with human cases of disease, were selected to generate WNV glycosylation mutant viruses via reverse genetics. Replication capacity and host competence of WNV glycosylation mutant viruses were compared *in vitro* and *in vivo* in American crows, house sparrows and *Culex* mosquitoes. The data demonstrated that N-linked glycosylation was not as crucial for WNV transmission and host competence as previously reported. Rather the amino acid identities of the glycosylation motif were more important in dictating WNV virulence phenotypes in both avian and mosquito host.

## Introduction

Since its identification in North America in 1999, West Nile virus (WNV) has caused large human encephalitis epidemics in the United States resulting in 48,183 reported human cases of neuroinvasive and non-neuroinvasive disease and 2,114 deaths to date (http://www.cdc.gov/westnile). First isolated in 1937 from a febrile patient in the West Nile district of northern Uganda[1], the virus is now known to circulate in Africa, Asia, Europe, North America, South America and Australia[2–4]. WNV is a member of the *Flaviviridae* family and is closely related to other human pathogens such as Japanese encephalitis (JEV), Saint Louis encephalitis and Murray Valley encephalitis viruses within the Japanese encephalitis virus (JEV) serocomplex[5, 6]. The natural reservoir/amplification hosts of WNV are avian species with *Culex spp*. mosquitoes serving as the primary vectors for enzootic transmission[4]. Humans and most mammals are incidental hosts in the natural transmission cycle as they do not develop sufficiently high viremias to be infectious for mosquitoes.

WNV has a single-stranded positive-sense RNA genome that is transcribed and translated in one open reading frame[5]. There are three structural proteins, capsid (C), pre-membrane (prM), envelope (E), and seven nonstructural (NS) proteins NS1-NS2A-NS2B-NS3-NS4A-NS4B-NS5 that are translated and released after host and viral protease cleavage in the cytoplasm[5]. The prM-E proteins influence efficiency of virus infection, viral particle maturation and virus release[7]. The prM is cleaved from the E protein during virus replication and assembly, allowing the E proteins to dimerize and create a lattice-like structure around mature WNV virus particles during viral maturation and release[8]. The E protein facilitates cell attachment, allowing virus entry into the cell via the endosomal pathway[5]. Both the prM and E proteins contain N-linked glycosylation sites; however, only the prM N-linked glycosylation site is highly conserved among WNV isolates[7].

The WNV E protein N-linked glycosylation motif (asparagine-tyrosine-serine/threonine; N-Y-S/T) lies between amino acid (aa) positions 154–156 and exists in forms predicted to be glycosylated and not glycosylated with the site completely absent in some strains due to a four amino acid deletion across this motif[9–11]. The N-linked glycosylation motif is present in most WNV strains that have been isolated during significant outbreaks of human disease both in North America and globally (Table 1)[7, 12–14]. Glycosylation of the WNV E protein has been previously implicated as an important viral genetic element for flavivirus virulence and pathogenesis and has been associated with enhanced growth in mammalian, avian and mosquito cells[7], and a murine neuroinvasive phenotype[15–18] with higher viremia and increased avian pathogenicity[19–21]_._ WNV virus-like particles (VLPs) with mutations that prevent glycosylation at the envelope motif, in contrast, have been associated with reduced particle production in mosquito, mammalian and avian cells however, VLPs lacking envelope glycosylation exhibited higher infectivity rates in mosquito cells compared to VLPs with glycosylated E proteins[7]. In other studies, a non-glycosylated NYI mutant virus demonstrated reduced transmissibility following oral exposure of *Culex pipiens* and reversion at the glycosylation motif was commonly identified in saliva samples of the orally exposed *Cx*. *pipiens*. Similarly, oral infectivity of the parental glycosylated WNV was found to infect *Cx*. *quinquefasciatus* more efficiently than the NYI mutant [22, 23]. Other flaviviruses such as Zika virus (ZIKV) also exhibit lower oral infectivity and vector competence in *Aedes aegypti* when the N-linked glycosylation was ablated from the envelope protein via mutagenesis[24, 25].

**Table 1:** Summary of West Nile virus strains including origin of isolation, glycosylation status and associated amino acid motif.

Isolate Name	Lineage	Year	Location	Origin	Glycosylation status	Amino Acid Motif
**TM171-03-pp1**	1a	2003	Mexico (Tabasco)	Raven	(-)	NYP
**LEIV-Vlg00-27924**	1a	2000	Russia (Volgograd)	Human	(+)	NYS
**NY99-flamingo-382-99**	1a	1999	USA (New York)	Flamingo	(+)	NYS
**IS-98**	1a	1998	Israel	Stork	(+)	NYS
**Italy.1998.Equine**	1a	1998	Italy	Horse	(+)	NYS
**KN3829**	1a	1998	Kenya (Rift Valley)	*Culex univittatus*	(+)	NYS
**RO97-50**	1a	1996	Romania	*Culex pipiens*	(+)	NYS
**ARD78016**	2	1990	Senegal	*Aedes vexans*	(-)	(---)
**Eth76**	2	1976	Ethiopia	Bird	(-)	SYS
**D00246**	1b	1960	Kunjin (MRM61C)	*Culex annulirostris*	(-)	NYF
**Eg101**	1a	1951	Egypt	Human	(-)	NYP

Many of the newly emergent and more virulent WNV strains, including the North American isolates, possess the E protein N-linked glycosylation motif (NYS) [16, 17, 19]. Previous reports have focused on preclusion of E protein glycosylation by mutagenizing NYS to IYS and AYS both of which have not been associated with any WNV isolate[22, 23]. However, the effect of naturally occurring WNV amino acid polymorphisms on maintenance of transmission cycles has not been investigated. Four glycosylation motif sequences found in naturally occurring isolates from North America, Australia, Europe and Africa (NYP, NYF, SYS, KYS, A’Del)[9, 26, 27] were reverse engineered and infectious clone-derived viruses generated. An additional WNV glycosylation mutant virus (NYT) was generated that was designed to alter the amino acid at E-156 while maintaining a glycosylation competent motif. An SYP mutant was generated as a double mutant in order to serve as a control, where both the first (E-154) and third amino acid (E-156) residues were mutagenized. Avian host, vector competence and viral growth capacity of these WNV infectious clone-derived glycosylation mutant viruses were assessed *in vitro* and *in vivo* in American crows (AMCRs) and house sparrows (HOSPs) and in *Cx*. *quinquefasciatus* and *Culex tarsalis*, respectively.

## Materials and methods

### Cells

C6/36 (*Aedes albopictus)* and Vero cells were maintained in Dulbecco’s Modified Eagle’s Medium (Gibco, Invitrogen, Carlsbad, CA) and duck embryonic fibroblast cells (DEF) were maintained in Eagle’s Minimum Essential media (EMEM, ATCC) with 10% heat-inactivated fetal bovine serum and 100U/mL and 100ug/mL of Penicillin/Streptomycin, respectively, at 28°C (mosquito cells) and 37°C for vertebrate cells. HD11 chicken monocytes were kindly provided by Dr. Kirk Klasing (UC Davis) and were maintained in RPMI 1640 media at 37°C. Vero cells were used for all plaque assays in this study.

### Viruses

Assembly and rescue of the WNV infectious clone derived virus (WNV.IC) has been described previously[28]. Site-directed mutagenesis primers (Table 2) were designed based on the glycosylation motif of the different WNV isolates (Table 1). Glycosylation mutant WNV plasmids were generated using a QuikChange site-directed mutagenesis kit (Invitrogen, Carlsbad, CA). To assemble the infectious clones, the viral genomic cDNA was digested out from a bipartite-plasmid system, ligated, *in vitro* transcribed and viral genomic RNA transfected into BHK cells as described previously[28]. The SYP and A’DEL (this mutant virus lacks the E αA’ helix (A’DEL; ΔE157-E160)) viruses were generated subsequently to assess the effect of mutating both the first and third amino acids in the glycosylation motif and to reduce the potential for genetic reversion at the glycosylation motif for *in vivo* studies and to assess the infectivity of a virus lacking the E154-156 motif, respectively. All viruses were harvested at 3 days post-transfection after observation of cytopathic effect (CPE). Full-length sequencing of the rescued viruses was performed to ensure the introduction of appropriate mutations and that extraneous mutations were not introduced. All viruses were titrated on Vero cells and plaque diameters measured (mm) from five representative plaques for comparison.

**Table 2:** Summary of West Nile virus infectious cDNA clone derived glycosylation mutant viruses that were generated.

Parental Virus^α^	Nucleotide Substitutions made to parental WNV.IC^β^	Rescued WNV Glycosylation Mutant Virus	Amino Acid Motif	Amino Acid Translation^β^	Predicted glycosylation status
**WNV.IC (NYS)**	AAT-TAC-TCC	WNV.IC	NYS	Asn- Tyr- Ser	(+)
	AAC-TAC-**A**CC	WNV.NYT.IC	NYT	Asn- Tyr- **Thr**	(+)
	AAC-TAC-**C**CC	WNV.NYP.IC	NYP	Asn- Tyr- **Pro**	(-)
	AAT-TAC-**TT**C	WNV.NYF.IC	NYF	Asn- Tyr- **Phe**	(-)
	A**G**C-TAC-TCC	WNV.SYS.IC	SYS	**Ser**- Tyr- Ser	(-)
	A**G**C-TAC-**C**CC	WNV.SYP.IC	SYP	**Ser**- Tyr- **Pro**	(-)
	AA**A**-TAC-TCC	WNV.KYS.IC	KYS	**Lys**- Tyr- Ser	(-)
	AAT-TAC-TCC	WNV.A’DEL.IC	A’DEL		(-)

^α^ The N-linked NYS (AAT-TAC-TCC) glycosylation motif of the parental WNV.IC envelope protein was mutagenized to generate seven new WNV glycosylation mutant viruses that are described in the third column.

^β^ Nucleotides and/or amino acids that were substituted are highlighted in bold and underlined.

### Virus passaging

In order to assess the stability of the E protein N-linked glycosylation motif in cell culture, serial passaging was performed in C6/36 cells. All viruses were passaged five times in C6/36 cells. Cells were initially inoculated in triplicate at an MOI of 0.01. Media was removed, virus was added and allowed to adsorb for one hour. After the absorption period, cells were washed twice with DPBS, after which fresh media was added. Cells were allowed to incubate for 7 dpi., after which supernatant was harvested, diluted 1:10 and added to a new flask of cells. This process was repeated for five passages. All viruses were harvested at 7 days post-infection (dpi) at which point RNA was extracted using a Qiagen Viral RNA extraction kit (Qiagen, Valencia, CA). Reverse transcription was performed using primer WNV4129R (5’ TTGAGGCTAGAGCCAAGCATAGCAG 3’) and Superscript II (Invitrogen, CA). Generated cDNA was diluted 100-fold and then used in PCR reactions with *Pfu* Turbo polymerase (Invitrogen, Carlsbad, CA) and primers WN128F (5’GCCGGGCTGTCAATATGCTAAAAC 3’) and WN2506R (5’ GCTCTTGCCGCTGATGTCTATG 3’). Amplicons were sequenced using primer WN1797R (5’ ATGACCCGACGTCAACTTGACAGTG 3’).

### Endoglycosidase digestion of envelope protein

In order to confirm the glycosylation status of the WNV mutants, Vero cells were inoculated with the parental and mutant viruses at an MOI of 1. At 50 hours post-inoculation (hpi), supernatant was removed and cells were lysed. Cell lysates were treated with 500 units of Endoglycosidase F enzyme (New England Biolabs), an amidase that cleaves the N-linked glycosylation moiety from the glycoprotein. Briefly,10 μg of cell lysate was denatured with 1X Glycoprotein Denaturing Buffer (0.5% SDS, 40mM DTT) at 100°C for 10 minutes. Cell lysate was then treated with glycoBuffer 2, NP-40 and Endoglycosidase F(+) after which the mix was incubated for 1 hour at 37°C. The samples (1 μg) were separated electrophoretically on a 4–20% reducing SDS gel. Separated proteins were electroblotted onto nitrocellulose membranes and immunostained with anti-WNV E mAb 3.67.

### *In vitro* growth kinetics

Vero, HD11, DEF and C6/36 cells were inoculated in triplicate with each virus at an MOI of 0.1. Viral titers were determined via a standard plaque assay on Vero cells and these titers were used to calculate MOIs for all *in vitro* growth kinetics experiments. Media was removed from the cells and 200μL of diluted virus was added to each well after which the cells were allowed to incubate with the virus at 28°C (C6/36 cells) or 37°C (all other cell lines) for one hour. After the incubation, cells were washed twice with DPBS and fresh media added to each well. The plates were incubated at the designated temperature (including up to 44°C for high temperature DEF cell assessments) through 4 dpi. 50μL of supernatant aliquots were harvested every 24 hours from each well and added to 450μL of DMEM supplemented with 20% heat-inactivated FBS. Samples were stored at -80°C until processed. Growth kinetics for each virus was determined by standard plaque assay on Vero cells. Viral titers were averaged, reported in log-based plaque-forming units/ mL and plotted by hpi.

### pH sensitivity assay

The parental strain and WNV glycosylation mutants were diluted to 10^5^ PFU/well and exposed to varying pH ranges (5.8–7.0) at 0.2 pH unit increments in triplicate for 10 minutes at which point the pH was brought to a neutral level (pH~7.4) by the addition of sterile PBS. Viral supernatants in the pH-neutralized buffer were 10-fold serial-titrated by plaque assay.

### Avian experimental infection studies

Wild AMCRs were captured by cannon nets in Bellvue, Colorado (U.S. Fish and Wildlife Scientific Collecting Permit number MB-032526). House sparrows were trapped by Japanese mist nets in Bakersfield, California. In order to confirm that AMCRs and HOSPs had not previously been exposed to WNV or another endemic flavivirus, St. Louis encephalitis virus (SLEV), birds were bled prior to inoculation and serum tested by plaque reduction neutralization assays (PRNTs) against these two viruses as previously described[29]. Detection of specific neutralizing antibodies within the sera of any AMCR or HOSP to either SLEV or WNV excluded the bird for use in *vivo* experiments. All AMCRs/HOSPs were held for at least two weeks prior to viral inoculation for cage adaptation and quarantine. 6–8 AMCRs were subcutaneously inoculated with 1,500 PFU of each virus (NYS, NYT, NYP NYF, SYS and KYS). Six HOSPs were similarly inoculated with 1,500 PFU of each WNV glycosylation mutant, including a mutant lacking the E αA’ helix (A’DEL; ΔE157-E160). All AMCRs and HOSPs were examined for signs of disease twice daily for 14 days following inoculation and bled once daily from 1 to 7 dpi to assess viremias. Viral RNA was extracted from serum samples at peak viremias and from brain tissue of AMCRs that had succumbed to infection and sequenced over the N-linked glycosylation motif to identify potential compensatory mutations in the E154-156 or surrounding regions using the method mentioned previously.

### Vector competence assessments

Four to seven-day-old *Culex quinquefasciatus* (Sebring strain) and *Culex tarsalis* (BFS), reared at 28°C, 16:8 (L: D) photo cycle with 5% sucrose solution, were used for vector competence assessments. The Sebring strain was originally collected in 1988 from Sebring County, Florida[30]. The *Cx*. *tarsalis* Bakersfield Field Station (BFS) strain was established from Bakersfield, Kern County, California and had been in colonization since 1952[31]. Seven groups of 100 female *Cx*. *quinquefasciatus* were sugar-starved for 24 hours prior to the feed. Viruses (NYS, NYT, NYP, NYF, SYS, SYP, KYS) were diluted to 7 log_10_ PFU/mL and mixed with defibrinated chicken blood (Colorado Serum Company, Denver, CO) at a 1:1 ratio. Each group of mosquitoes was exposed to 2 mL of the virus: blood mix using a Hemotek feeding unit (Discovery Workshops, Accrington, UK,). After one hour of feeding, fully engorged females were held at 28°C with 5% sucrose under a 16:8 (L:D) photoperiod through 14 days post-exposure (dpe). The A’DEL virus did not produce viral titers higher than 6.3 log_10_ PFU/mL at the time of this study. Therefore, a separate experiment was performed where the NYS virus was also diluted to 6.7 log_10_ PFU/mL for comparison and artificial oral infections with A’DEL and NYS were performed as described above. At 14 dpe, 25 mosquitoes were removed from each virus group, anesthetized by exposure to triethylamine, legs removed and saliva collected by capillary tube as previously described[32]. The hind leg was removed from each mosquito and was placed in 0.5mL of diluent along with a BB (Crossman Corporation, NY). Mosquito bodies were collected after salivation was completed and stored in 0.5mL of diluent with a BB. Mosquito bodies, legs and saliva were stored at -80°C until titrated by plaque assay. Only 0.2mL of each sample was 10- fold serially titrated and viral titers were calculated as log_10_ PFU/mL. A direct 2.5-fold conversion can be applied to identify titers from whole bodies, legs and expectorants, respectively.

Bodies were homogenized using a mixer mill at 24 cycles/sec for 2 minutes. Homogenates were clarified via centrifugation for 5 min at 5,000 x g and assayed for the presence of virus by plaque titration on Vero cells. Infection rates were calculated as the number of virus-positive bodies as a percentage of the total number of mosquitoes assayed at 14 dpe. Dissemination rates were calculated as the number of virus-positive legs as a percentage of virus-positive bodies. Saliva samples from mosquitoes that exhibited virus positive legs were centrifuged at 5,000 x g for 5 min and titrated on Vero cells as described above. Transmission rates were calculated as the percentage virus-positive saliva as a percentage of virus-positive legs. *Culex tarsalis* were also exposed to artificial infectious blood meals and were processed in the same manner as the *Cx*. *quinquefasciatus*.

### Intrathoracic inoculations

One hundred female *Cx*. *quinquefasciatus* were individually intrathoracically inoculated with 0.14μL of 6 log_10_ PFU/mL (~140 PFU) of the parental NYS virus and each of the mutant viruses (NYT, NYP, NYF, SYS, SYP and KYS). Following inoculation, mosquitoes were held at 28°C with 5% sucrose through 7 dpe under a photoperiod of 16:8 (L: D). At 7 dpe, 50 mosquitoes exposed to each virus were anesthetized as described above. Saliva and bodies were collected, stored and titrated as described previously. Infection rates were calculated as described above. The number of virus-positive saliva samples were expressed as a percentage of virus-positive bodies to calculate transmission rates

### Statistical analysis

For all negative samples, the LOD value was used for all statistical evaluations. One-way ANOVA tests were used to assess differences in mean peak viremia in AMCRs and HOSPs as well as between titers at various time points between mutant viruses in Vero, DEF, C6/36 and HD11 cells and plaque sizes. Tukey’s HSD adjustment for multiple comparisons were utilized for assessing mean differences. Pair-wise Fisher’s exact tests were used to analyze differences in infection, dissemination and transmission rates of each mutant virus compared to the parental glycosylated NYS WNV in mosquitoes.

### Ethics statement

California and Colorado birds were collected, housed, transported and inoculated under the following approved permits and protocols: i) University of California, Davis, Institutional Animal Care and Use Committee (IACUC) protocols 12876 and 12880 ii) Colorado State University, Institutional Animal Care and Use Committee protocol 10-2078A iii) USGS Master Station Banding Permit 22763 iv) State of California Scientific Collecting Permits v) Federal Permit MB082812 vi) BUA 0554 by the University of California, Davis, Environmental Health and Safety Committee, and USDA Permit 47901 vii) IACUC 10-2078A in the Animal Disease Lab at Colorado State University.

## Results

### Generation and characterization of WNV mutants

**Fig 1:**
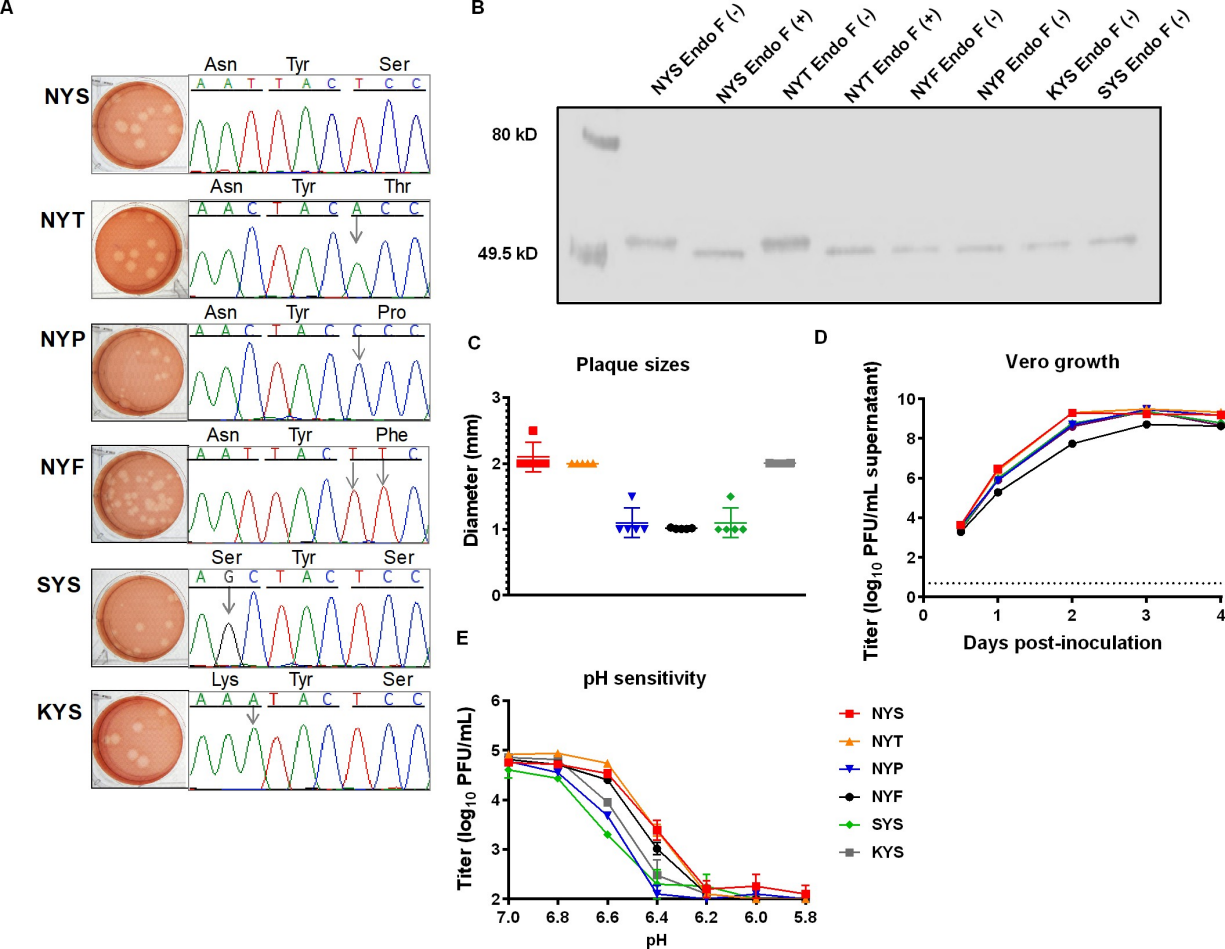
*In vitro* characterization of WNV glycosylation mutant viruses. (A) Plaque morphology of WNV mutants on Vero cells at 3 dpi and sequenced across the E154-156 motif. Arrows designate genetic loci modified by sire-directed mutagenesis. (B) Endoglycosidase digestion (Endo F) was used to assess presence/ absence of WNV envelope protein glycosylation. Purified E proteins were digested with Endoglycosidase F, Endo F (+), or left undigested Endo F (-). Migration rates of the E proteins were determined by resolving the viral proteins on a 4–20% SDS-PAGE gel and performing a Western blot with the WNV E-glycoprotein specific monoclonal antibody, 3.67G. (C) Plaque diameters were measured (mm) from five representative plaques from each glycosylation mutant virus (D)Virus growth was assessed in Vero cells with the WNV parental virus and mutants at an MOI of 0. 1. The Limit of Detection (LOD) shown by dashed line. (E) The parental strain and WNV glycosylation mutants were diluted to 10^5^ PFU/well and exposed to varying pH ranges (5.8–7.0) at 0.2 pH unit increments in triplicate for 10 minutes. The pH was immediately brought to a neutral condition (pH 7.4) by the addition of sterile PBS. Viral titers of pH- treated samples were determined via plaque assay, reported in log_10_ PFU/mL and plotted as a function of the pH treatment.

WNV mutants (Table 2) were harvested from transfected BHK-21 supernatants after observation of cytopathic effect at day three post-transfection. Rescued mutants were titrated and full-length Sanger sequencing performed to confirm the incorporation of the correct mutations and that spurious mutations were not inadvertently introduced (Fig 1A). A Western blot was performed to assess the glycosylation status of E viral proteins purified from Vero cells infected with the parental and mutant viruses. The E proteins of endoglycosidase untreated control NYS [NYS EndoF(-)] and NYT [NYT EndoF(-)] viruses ran at >50 kDa. (Fig 1B). After treatment with endoglycosidase F, E proteins of both NYS and NYT viruses increased in mobility and migrated at <49 kDa (Fig 1B), indicating de-glycosylation of NYS and NYT via endoglycosidase treatment. Mobility of NYS and NYT endoglycosidase-treated E proteins was indistinguishable from the untreated NYF, NYP, KYS and SYS E proteins, indicating that the nucleotide substitutions to create NYF, NYP, KYS and SYS successfully ablated glycosylation of the E protein[7, 10, 22, 27, 33]. Plaques from NYS, NYT, KYS were significantly larger than (p<0.05) NYP,NYF and SYS plaques (Fig 1C).

### Differential *in vitro* growth of WNV mutants

Growth profiles of glycosylation competent viruses (NYS, NYT) were indistinguishable in Vero cells (Fig 1D), with both NYS (9.3 log_10_ PFU/mL) and NYT (9.3 log_10_ PFU/mL) viruses reaching peak viral titers at 2 dpi (p>0.1). The nonglycosylated (SYS, NYP and KYS) mutants exhibited mean viral titers that were at least 5-fold lower than the glycosylated parental virus at 2 dpi (p<0.005) and reached indistinguishable mean peak viral titers compared to the glycosylated NYS virus by 3 dpi (p>0.5). The NYF mutant showed a more significant growth restriction in Vero cells, with an ~50-fold reduction in titer compared to the NYS virus in Vero cells at 2 dpi (p<0.0005). Furthermore, the mean peak titer at 3 dpi was also at least 5-fold lower than either the NYS or NYT viruses (p<0.009).

Since glycosylation of the E154-156 motif has been associated with differential protein stability that could be critical under low pH conditions in which the E protein is exposed within the endocytic vesicle, the assessment of infectivity was performed under an acidic pH range[34] (Fig 1E). Both glycosylated variants (NYS/NYT) maintained infectious mean viral titers that were within 3-fold of the pretreatment mean viral titer at pH ranges of 6.6–7.0. The non-glycosylated NYF mutant similarly exhibited a 3-fold drop in mean viral titer within this same pH range. In contrast, the NYP, KYS and SYS mutants all showed approximately 10-fold reductions in infectious titers at pH 6.6 versus 7.0.

**Fig 2:**
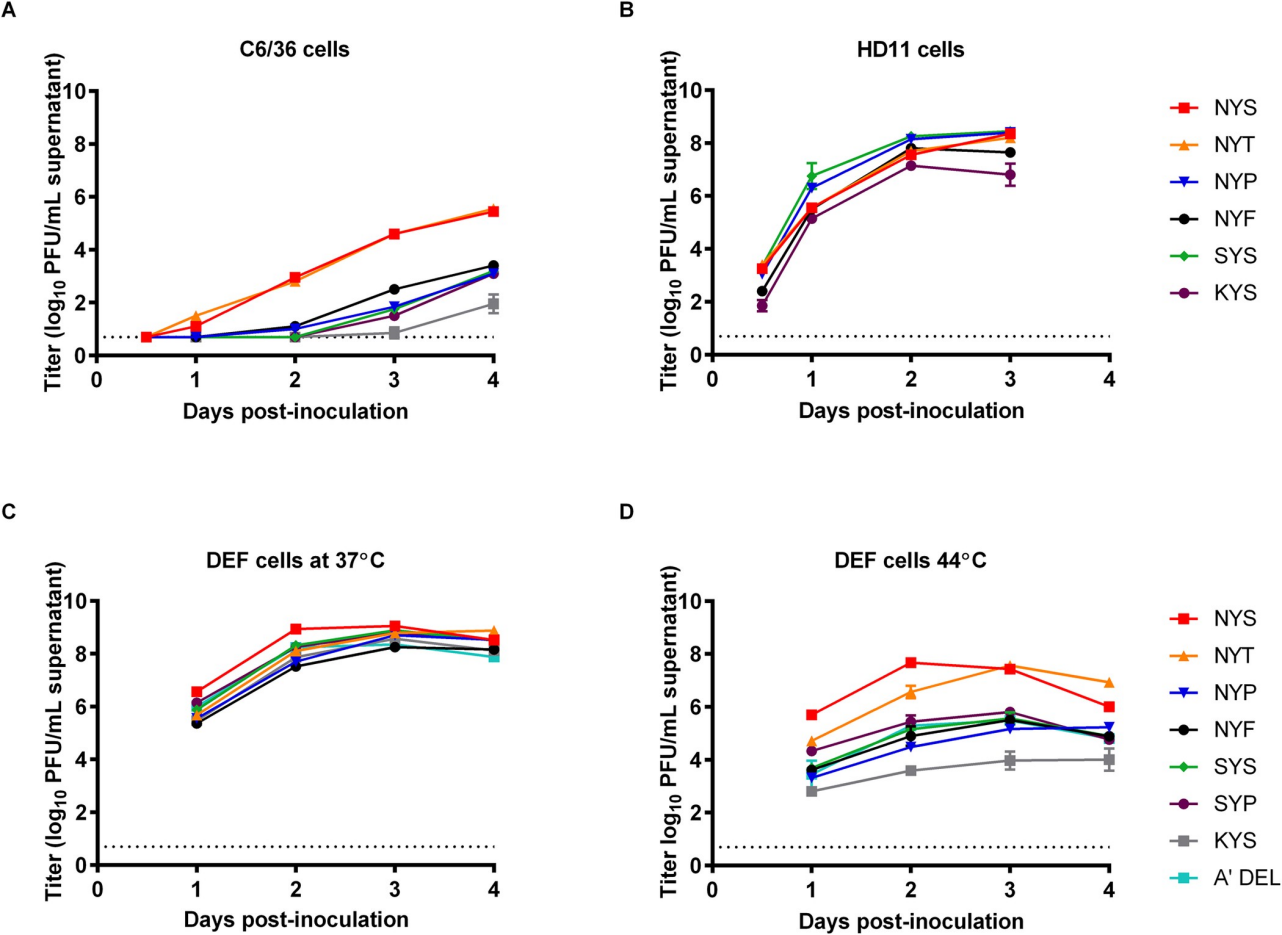
Virus growth of parental and WNV glycosylation mutant viruses. (A) mosquito cells (C6/36) (B) HD11 cells (C) DEF cells at 37°C and (D) DEF cells at 44°C inoculated at an MOI of 0. 1. Supernatants were collected at 12, 24, 48, 72 and 96 hpi (C6/36 and HD11 cells) and 24, 48, 72 and 96 hpi (DEF cells), diluted 1:10 and stored until titration using a standard plaque assay. Viral titers were reported in log_10_ PFU/mL and plotted against hours post-inoculation. Viral titers were statistically analyzed via a one-way ANOVA and asterisks represent significant differences with a p-value of <0.05. LODs are shown by dashed lines.

Similar to results in Vero cells, growth of the glycosylated viruses, NYS and NYT, in C6/36 cells was statistically indistinguishable, reaching daily mean titer of 5.5 ± 0.1 log_10_ PFU/mL by 4 dpi (Fig 2A). Both NYS/NYT WNVs produced significantly higher titers (p<0.05) than NYP, NYF, SYS, SYP and KYS, between 1–4 dpi. All nonglycosylated mutants exhibited a delay in the detection of virus until 2 dpi. From 2–4 dpi, NYP, NYF, SYS, SYP and KYS titers were significantly lower (p<0.05) than NYS and NYT by 100-1000-fold. Titers for KYS were initially detected at 3 dpi and exhibited the most significantly retarded (p<0.05) growth in C6/36 cells in comparison with the other WNV mutants. Serial passaging was performed to assess genetic stability of the E protein glycosylation motif. After five serial passages in C6/36 cells, the NYS, NYT, NYP, NYF, SYP and KYS viruses were found to have retained the introduced mutations. One replicate of the third passage of SYS showed a mixed population phenotype at both positions E-154 and E-156. The E-154 codon showed nucleotide changes from AGC (Ser) to AAC/T (Asn)/A(Lys) and codon E-156 showed changes from TCC (Ser) to T/CCC (Ser/Pro).

Since monocyte/macrophage cell populations have been implicated in the amplification and dissemination of WNV in avian infections[35, 36], mutants were compared in cultured avian monocytes (HD11 cells) (Fig 2B). The NYS/NYT viruses demonstrated statistically indistinguishable growth while the NYF mutant exhibited a 7-fold lower viral titer (p<0.05) at the earliest time point (0.5 dpi) and the mean peak titer was 3-fold lower than that of NYS. The KYS mutant demonstrated the most restricted phenotype with a 16-fold lower mean peak viral titer compared to NYS virus (p<0.05). In contrast, the non-glycosylated NYP/SYS mutants generated higher mean viral titers 6.3 and 6.8 log_10_ PFU/mL, respectively compared to 5.6 log_10_ PFU/mL at 1 dpi; however, the mean peak titers at 3 dpi were indistinguishable to that of NYS.

Temperature sensitivity in avian cells has previously been associated with differential avian host competence[37]. As such, DEF cells growth phenotypes of the WNV mutants were assessed at 37°C/44°C (Fig 2C and 2D). All WNVs grew to titers >8 log_10_ PFU/mL at 37°C; however, differences in growth were apparent at 44°C. Glycosylated viruses manifested mean titers of >7 log_10_ PFU/mL supernatant at the elevated temperature; however, NYP, NYF, SYS, SYP and A’DEL mutants all grew to mean peak viral titers of only approximately 5.5 log_10_ PFU/mL supernatant at 44°C, demonstrating a >50-fold higher temperature sensitivity than NYS/NYT. In contrast, KYS failed to grow above 4 log_10_ PFU/mL supernatant at the elevated temperature, demonstrating >30,000-fold lower titer at 44°C than at 37°C compared to approximately a 100-fold and 1000-fold temperature sensitivity phenotype of NYS/NYT and alternative E-154-56 mutants, respectively.

### American crow host competence

**Fig 3:**
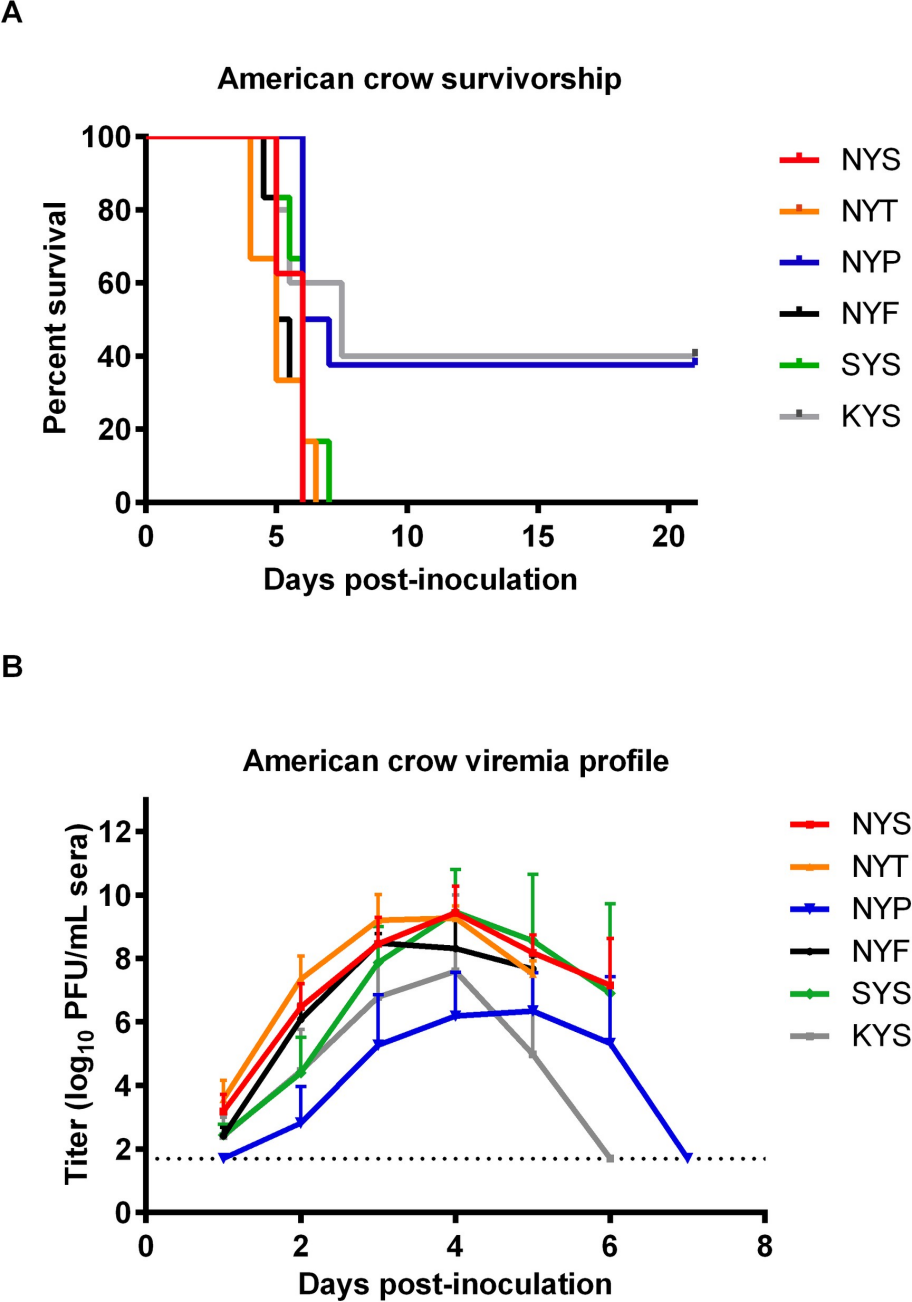
WNV glycosylation mutant virus growth and survivorship in American crows (AMCR). Seven AMCRs were inoculated with 1,500 PFU of parental WNV and mutants. Graphs represent (A) survivorship and (B) mean daily peak viremias (error bars denoted standard deviations from the mean) in log_10_ PFU/mL sera. LODs are shown by dashed line.

Since AMCRs are highly susceptible to WNV[29, 38], the impact of the various WNV mutants on avian survivorship was assessed. All crows inoculated with NYS, NYT, NYF and SYS mutants succumbed to infection at indistinguishable rates over a 6–7 dpi time period (Fig 3A). Crows inoculated with NYS, NYT, NYF and SYS viruses all developed acute viremias >8.5 log_10_ PFU/mL sera (Fig 3B). In contrast, NYP and KYS mutants both resulted in only 60% mortality rates (Fig 3A) and exhibited attenuated viremia production (<7.5 log_10_ PFU/mL sera) (Fig 3B). No differences in peak viremia or viremia on any dpi were observed between the glycosylated NYS/NYT (Fig 3B). The non-glycosylated NYF/SYS mutants demonstrated no significant differences in the magnitude (p>0.9) or duration (p>0.09) of peripheral viremias compared to the NYS/NYT. TheNYP/KYS mutants, although demonstrating indistinguishable viremia duration compared to NYS/NYT (p>0.7) (Fig 3B), exhibited attenuated viremia production with mean peak viral titers 1,200-fold (p<0.008) and 68-fold (p<0.1) lower, respectively. Neither reversions nor compensatory mutations were identified in the E154-156 motifs observed from serum viral RNA sequenced from each bird at the time point with the highest viremia. Viral sequence from brain tissue of AMCRs that succumbed to infection also failed to demonstrate any genetic changes over the 500-nucleotide amplicon surrounding the E N-linked glycosylation motif when compared to the inoculum consensus sequences.

### Viremia profiles of WNV glycosylation mutants in HOSPs

**Fig 4:**
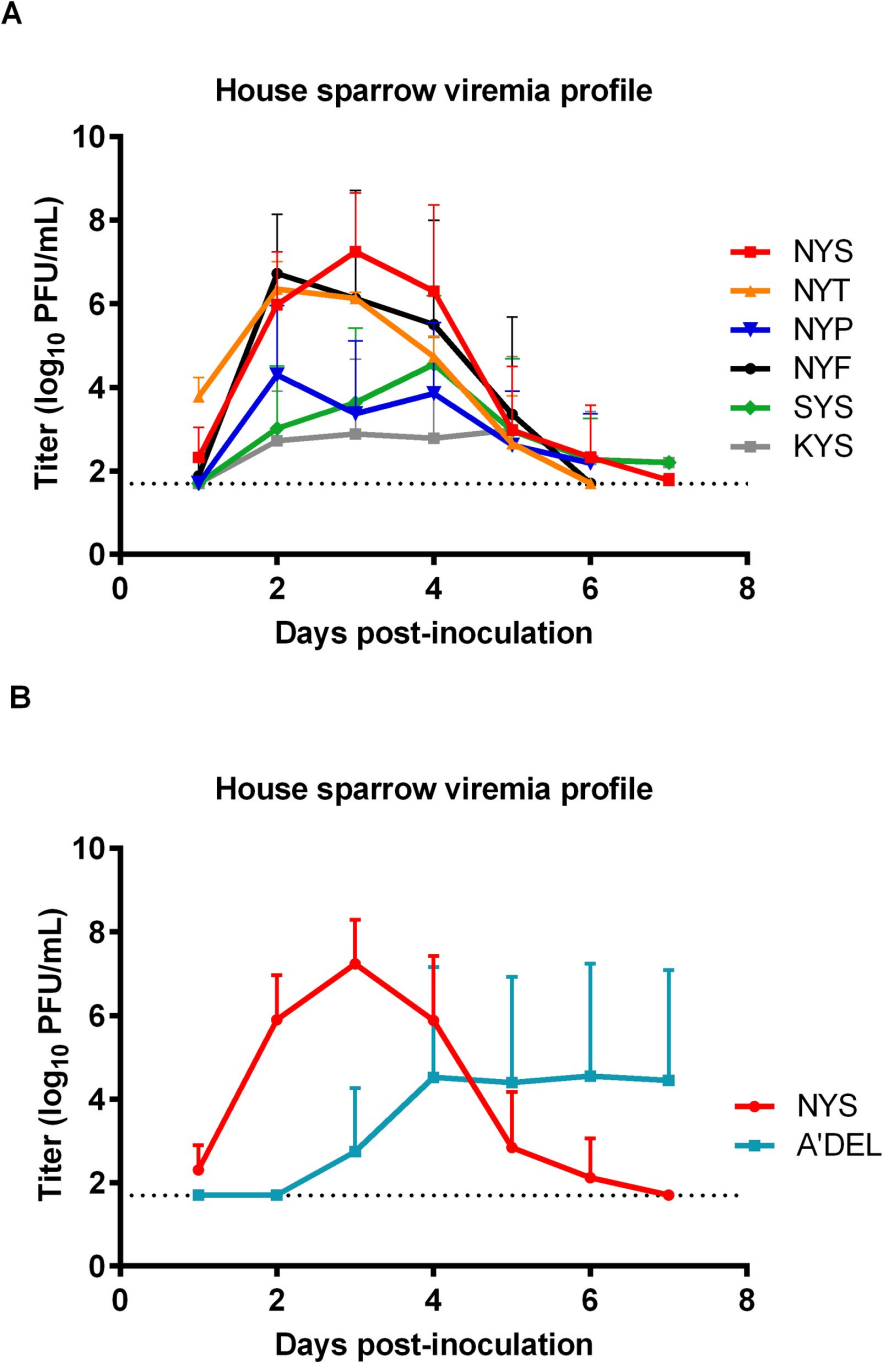
WNV glycosylation mutant virus growth and survivorship in house sparrows (HOSP). Viremia profiles of six HOSPs inoculated with 1,500 PFU of NYS and mutant WNVs. (A) Mean daily peak viremias (error bars denoted standard deviations from the mean) of HOSPs inoculated with NYS, NYT, NYP, NYF, SYS and KYS. (B) Mean daily peak viremias (error bars denoted standard deviations from the mean) of HOSPs inoculated with NYS and A’DEL mutant. LODs are shown by dashed lines.

The viremia response to the different mutants was evaluated in an alternative avian species, HOSPs (Fig 4A and 4B). In concordance with the AMCR viremia data, the NYF non-glycosylated mutant developed statistically indistinguishable mean peak viral loads compared to the glycosylated (NYS/NYT) viruses (p>0.1). In contrast, despite developing high viremias in AMCRs, the SYS mutant was debilitated in the HOSPs, producing a ~500-fold lower mean peak viral titer when compared to the NYS virus (p<0.05). The NYP mutant developed a mean peak viral load that was 91-fold lower but was not statistically significantly different from the NYS virus (p = 0.571). The KYS mutant mean peak viremia was 30,000-fold lower (p = 0.044) than NYS. The NYT mutant elicited a significantly higher initial viremia than the NYS virus at dpi 1 (p<0.01); however, no significant differences were observed from dpi 2–7. In a separate study, the viremia potential of the A’DEL mutant was assessed compared to the NYS parental virus. Two of the six HOSPs inoculated with the A’DEL mutant failed to generate detectable viremias during the course of the 1–7 dpi serum sampling (Fig 4B). A two-day delay in viremia onset in the four HOSPs that became viremic was observed with the NYS titers significantly higher than the A’DEL titers at dpi 1–3; however, the A’DEL birds subsequently developed significant viremias from 3–7 dpi and peak titers for individual birds showed no difference compared to NYS inoculated HOSPs (p = 0.252) (Fig 4B). Mortality rates were low in all groups and not significantly different between any virus infection group and thus have not been shown. Sequencing of HOSP serum samples was also not performed due to the aforementioned reason.

### Vector competence of WNV glycosylation mutants in *Culex* mosquitoes

**Fig 5:**
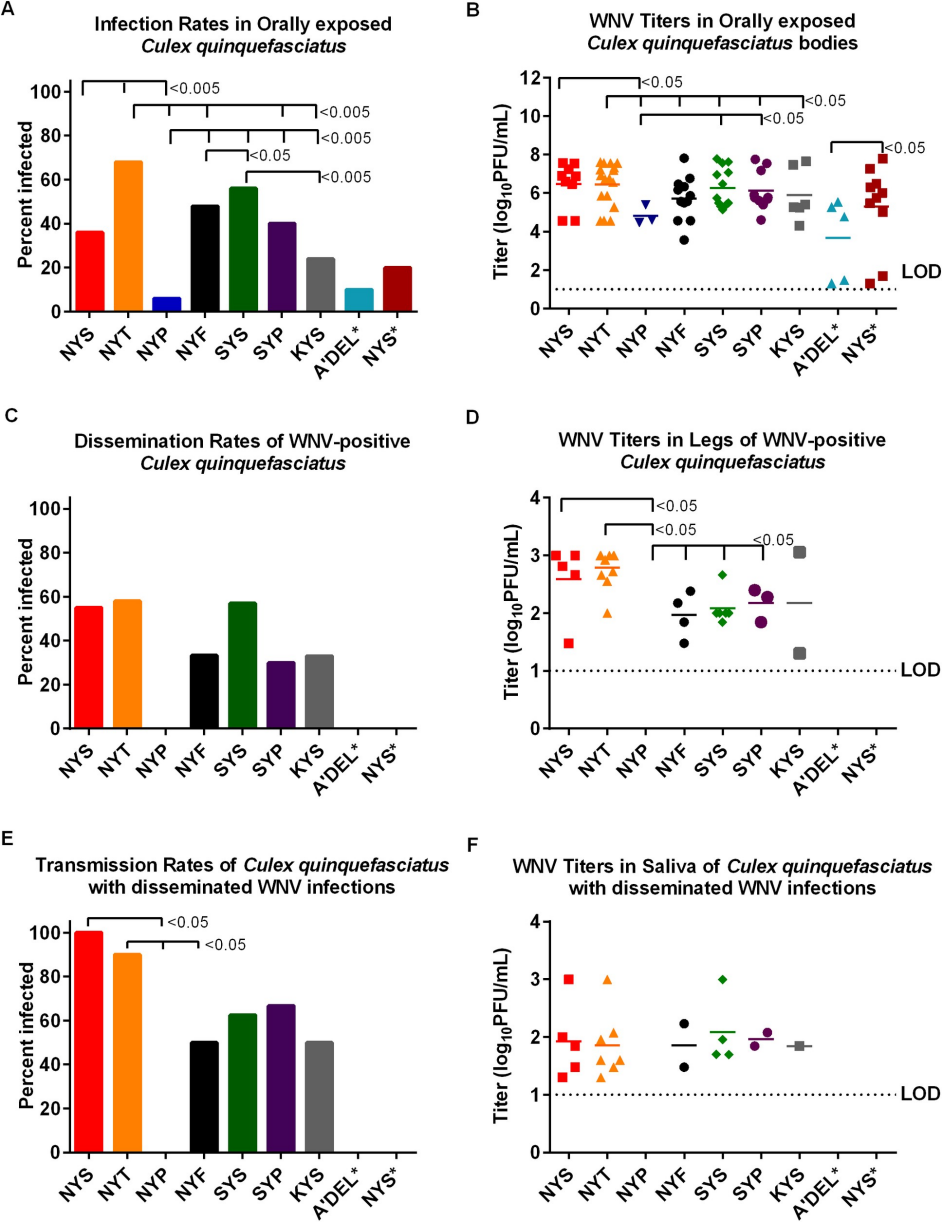
Vector competence assessment of WNV glycosylation mutant viruses in *Culex quinquefasciatus*. *Culex quinquefasciatus* were orally exposed to individual artificial blood meals containing 7 log_10_ PFU/mL of the WNV parental (NYS) or glycosylation mutant viruses (NYT, NYP, NYF, SYS, SYP, KYS) or 6.3 log_10_ PFU/mL of NYS and A’DEL (indicated as NYS* and A’DEL*). After an extrinsic incubation period of 14 days, bodies, legs and saliva were collected from 25 mosquitoes and titrated by plaque assay. The Figure panels demonstrate (A) infection rate (%) of *Cx*. *quinquefasciatus* bodies positive for virus as a function of exposed mosquitoes (B) viral titers of infected *Cx*. *quinquefasciatus* bodies (C) dissemination rate (%) of infected mosquitoes with virus-positive legs (D) viral titers in *Cx*. *quinquefasciatus* infected legs (E) percent of mosquitoes with disseminated infections that had virus-positive saliva (F) viral titers in mosquito saliva. Mean viral titers were analyzed via a one-way ANOVA. Infection, dissemination and transmission rates were analyzed using a pairwise Fisher’s exact test. Limit of detection (LOD) is shown by dashed lines.

*Culex quinquefasciatus* were orally exposed to WNV parental (NYS) and mutant viruses, NYT, NYP, NYF, SYS, SYP and KYS, in order to examine the effect of the variable glycosylation motifs on mosquito infectivity and subsequent transmissibility. Oral infectivity for the parental NYS virus (36%) was significantly lower (p<0.005) than that of the NYT mutant (68%) and significantly higher (p<0.005) than the NYP infection rate of 6% (Fig 5A). The NYT infection rate was significantly higher (p<0.005) than rates for NYP, NYF (48%), SYP (40%) and KYS (24%) mutant viruses. The infection rate for NYP was significantly lower (p<0.005) than all the other viruses. Among the remainder of the nonglycosylated viruses, the SYS (56%) infection rate was significantly higher than NYF (p<0.05) and KYS (p<0.005). Oral infectivity between A’DEL* (10%) and NYS* (20%), performed at a lower oral input titer due to the restricted growth of the deletion mutant (A'DEL) in cell culture, were not significantly different.

No significant differences in the mean viral titers of positive mosquito bodies were observed for *Cx*. *quinquefasciatus* orally exposed to NYS/NYT viruses; however, mean body titers of both NYT and NYS exposed *Cx*. *quinquefasciatus* were significantly higher (p<0.05) than NYP (Fig 5B). Mean NYT viral titers were also significantly higher (p<0.05) than NYP, NYF, SYS, SYP and KYS (Fig 5B). Among the nonglycosylated viruses, NYP exposed mosquitoes exhibited a significantly lower (p<0.05) mean viral load than SYS and SYP exposed mosquitoes (Fig 5B). Mean viral loads of A’DEL* were significantly lower (p<0.05) than NYS* (Fig 5B).

No disseminated infections (<LOD) in the three bodies positive for NYP were observed. There were no significant differences (p>0.05) in dissemination rates between any of the viruses for which dissemination was observed (Fig 5C). Disseminated infections were not observed for both A’DEL* and NYS* (Fig 5C). Mean viral titers in *Cx*. *quinquefasciatus* legs were not significantly different (p>0.05) between NYS/NYT (Fig 5D). No NYP leg titers were observed above the limit of detection. Using the LOD for NYP dissemination resulted in a significantly lower (p<0.05) titer compared to NYS, NYT, NYF, SYS and SYP leg titers. Transmission rates of NYT disseminated mosquitoes were significantly higher than those of NYP and NYF and transmission rate of NYS was significantly higher than NYP (Fig 5E)There were no other significant differences (p>0.05) in transmission rates among NYS, NYT, SYS, SYP and KYS (Fig 5E). There were no significant differences (p>0.05) in mean viral titers in the saliva (Fig 5F). When transmission rates were compared as a function of the total exposed mosquitoes, NYT exhibited a 36% transmission rate with a 20% transmission rate for NYS; however, this difference was not significant (p = 0.3451).

**Fig 6:**
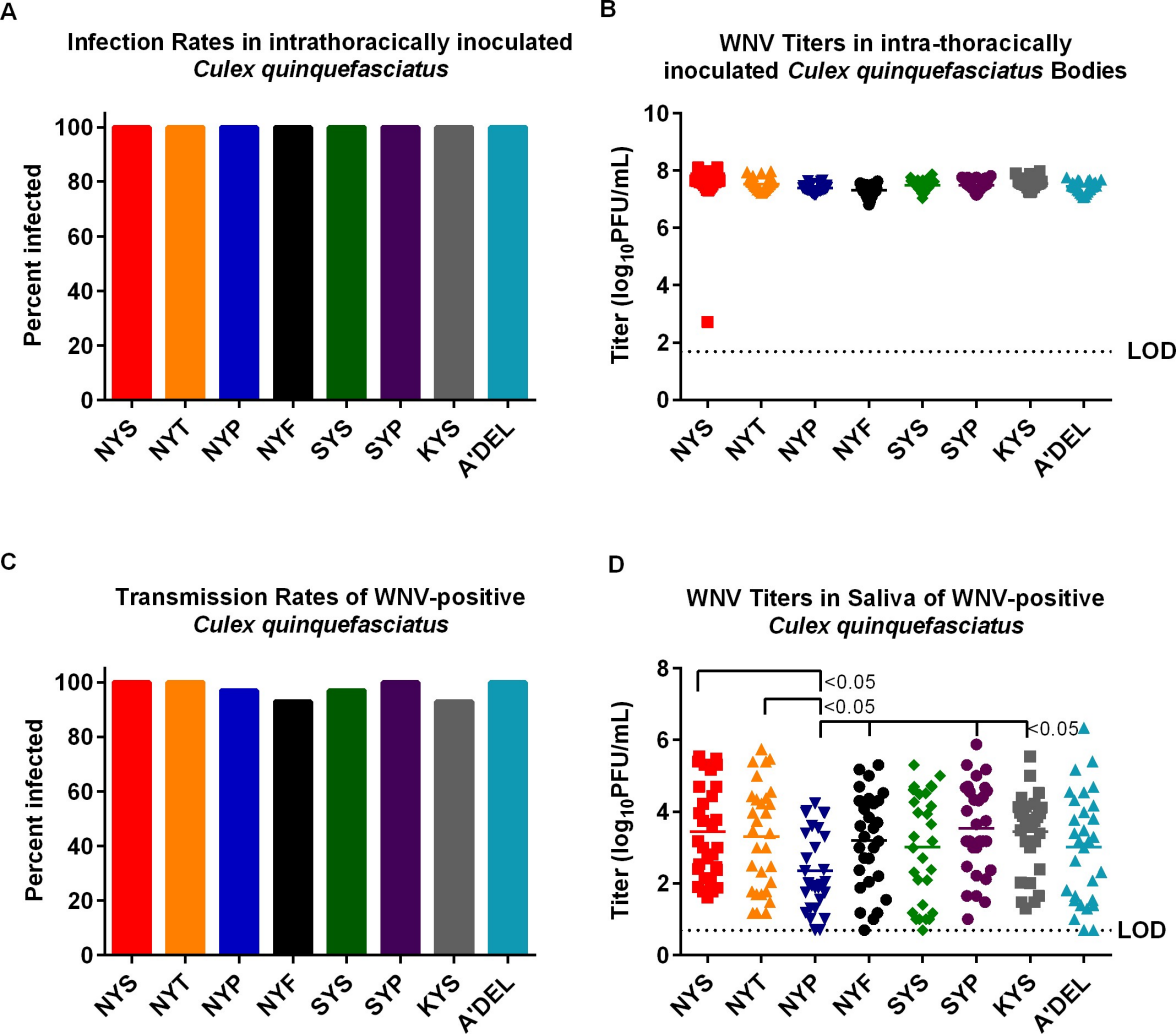
Viral growth in *Culex quinquefasciatus* intrathoracically inoculated with WNV glycosylation mutant viruses. *Culex quinquefasciatus* were intrathoracically inoculated with 0.14μL of 6 log_10_ PFU/mL of WNV parental and WNV glycosylation mutant viruses (NYT, NYP, NYF, SYS, SYP, KYS, and A’DEL). After an extrinsic incubation period of 7 days, bodies and saliva were collected from 25 mosquitoes, mixed with diluent, homogenized and titrated by standard plaque assay. Figures demonstrate (A) infection rate (%) in *Cx*. *quinquefasciatus* bodies (B) viral titers in *Cx*. *quinquefasciatus* bodies in log_10_ PFU/mL (C) transmission rate (%) in *Cx*. *quinquefasciatus* saliva (D) viral titers in *Cx*. *quinquefasciatus* saliva in log_10_ PFU/mL. Mean viral titers were analyzed via a one-way ANOVA. Infection and transmission rates were analyzed using a pairwise Fisher’s exact test. Limit of detection (LOD) is shown by dashed lines.

*Culex quinquefasciatus* were intrathoracically (IT) inoculated with NYS or mutant viruses, NYT, NYP, NYF, SYS, SYP, KYS and A’DEL to assess viral growth and transmission potential independent of the midgut infection barrier. No significant (p>0.05) differences were observed in infection rates (Fig 6A) or mean body titers (100% for all viruses; Fig 6B) for any WNV in IT-inoculated mosquitoes. No significant differences (p>0.05) were observed in transmission rates among the viruses (Fig 6C). Mean viral saliva titers for NYS inoculated mosquitoes were not significantly (p>0.05) different from NYT inoculated mosquitoes (Fig 6D); however, both NYS and NYT viruses had significantly higher mean saliva titers (p<0.05) than NYP. Mean saliva NYP viral titers were also significantly lower (p<0.05) than those of NYF, SYP and KYS infected mosquitoes (Fig 6D). The *in vitro* passaging data revealed the stability of the introduced mutations at E154-156 after five passages in C6/36 cells, therefore viral sequencing was not performed on any mosquito samples.

**Fig 7:**
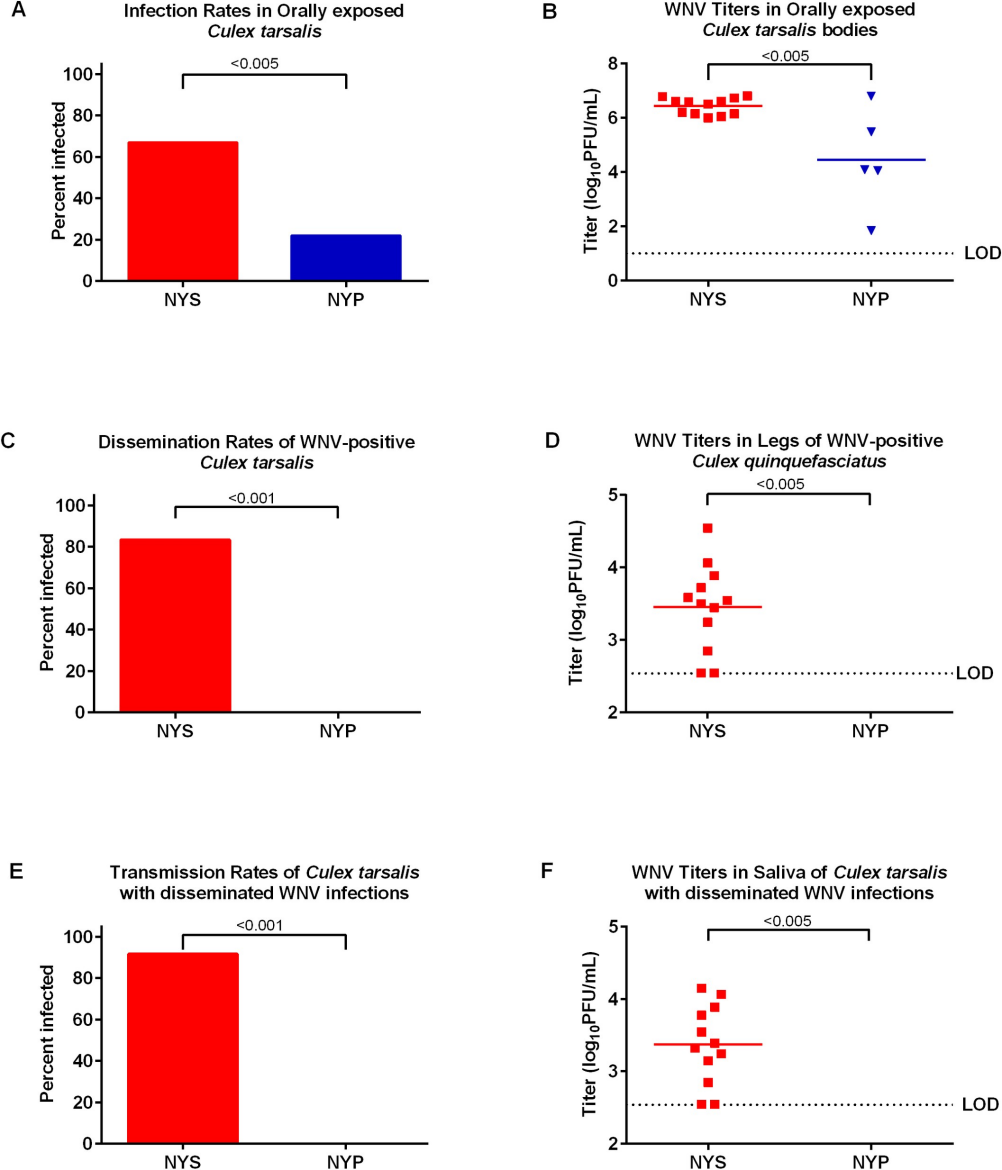
Vector competence assessment of WNV glycosylation mutant viruses in *Culex tarsalis*. *Culex tarsalis* were orally exposed to individual artificial blood meals containing 7 log_10_ PFU/mL of parental WNV (NYS) or WNV glycosylation mutant virus (NYP). After the extrinsic incubation period of 14 days, bodies, legs and saliva were collected from 25 mosquitoes, mixed with diluent, homogenized and stored until titrations using a standard plaque assay. Figures show (A) infection rate (%) in *Cx*. *tarsalis* bodies (B) viral titers of *Cx*. *tarsalis* bodies in log_10_ PFU/mL (C) dissemination rate (%) in *Cx*. *tarsalis* legs (D) viral titers of *Cx*. *tarsalis* legs in log_10_ PFU/mL (E) transmission rates (%) observed in *Cx*. *tarsalis* saliva (F) viral titers of *Cx*. *tarsalis* saliva in log_10_ PFU/mL. Mean viral titers were analyzed via a one-way ANOVA. Infection, dissemination and transmission rates were analyzed using a pairwise Fisher’s exact test. Limit of detection (LOD) is shown by dashed lines.

The NYP virus demonstrated the most attenuated infection, dissemination and transmission rates in *Cx*. *quinquefasciatus*. As such, the NYP mutant was assessed in an alternative mosquito species, *Cx*. *tarsalis*, in order to determine its relative effect in another important North American WNV enzootic vector species (Fig 7). Similarly, the infection (Fig 7A), dissemination (Fig 7C) and transmission rates (Fig 7E) for NYS were significantly higher (p<0.005, 0.001, 0.001, respectively) than those observed for NYP. Mean viral titers in NYS-positive mosquito bodies (Fig 7B), legs (Fig 7D) and saliva (Fig 7F) were significantly higher (p<0.005) than the mean viral titers for NYP-positive mosquito bodies, legs and saliva.

## Discussion

The N-linked glycosylation motif within the WNV E protein at position 154–156 has been implicated previously as an important molecular determinant associated with enhanced murine neuroinvasiveness, avian pathogenicity[15, 18–21] and infectivity and transmissibility in *Culex* mosquitoes[22, 23]. In this study, we investigated WNV viral replication, WNV-vector interactions, avian virulence and vector competence of variable amino acid polymorphisms at the E-154 or E-156 loci that resulted in predicted glycosylated (NYS, NYT) and non-glycosylated E proteins (NYP, NYF, SYS, SYP, KYS and A’DEL). We demonstrate the novel finding that the amino acid identity at either E-154 or E-156 modulated WNV viral growth, avian virulence and vector competence independent of the predicted glycosylation of WNV-E protein.

In previous reports, WNV mutants with abolished E-protein glycosylation such as NYF[27], QYS[7] and AYS[39] exhibited attenuated *in vitro* viral growth profiles in comparison to NYS whereas mutants representing NYS to IYS[22], NYE or NYP[19], were reported to have a minimal impact on viral growth in C6/36 cells. Other groups have reported that mutating NYS to QYS resulted in strikingly higher levels of infectivity in mosquito cells but lower virus production when compared with NYS[7]. These previous reports focused on ablation of glycosylation and did not specifically assess the effect of the actual amino acid identity at E-154/ E-156 on phenotype. The results presented herein have demonstrated that introduction of amino acid polymorphisms at either E-154 or E-156 differentially modulated WNV growth in C6/36 cells. Although many of the nonglycosylated WNVs showed an increased sensitivity to low pH, they all grew quite well in Vero, HD11 and DEF cells at 37°C. Interestingly, although temperature sensitivity at 44°C has been associated with lessened avian virulence phenotypes of other WNV variants/mutants[28, 37], many of the nonglycosylated WNV mutants (NYF and SYS) that were highly sensitive for growth at elevated avian temperatures *in vitro* demonstrated high viremia levels in AMCRs (NYF and SYS) or HOSPs (NYF). These data indicate that amino acid polymorphisms in this region of the E protein, although impacting temperature sensitivity do not necessarily alter avian host competence phenotypes. Previous work has demonstrated nonstructural genetic determinants to be positively correlated with both temperature sensitivity and avian host competence phenotypes[36, 40], indicating that structural determinants of temperature sensitivity observed herein could be dictated by factors unrelated to avian competence.

Unlike previous studies in which the growth of WNV mutants in AMCR monocytes predicted viremia potential and virulence[35], all viruses grew relatively well in chicken (HD11) monocytes herein, indicating fundamental differences in the relative utility for avian cells from alternative avian sources with lessened susceptibility (chickens) to WNV to model avian virulence phenotypes in highly susceptible birds (AMCRs). In previous reports, HD11 cells had demonstrated restricted growth for a Mexican isolate of WNV lacking a glycosylation motif (NYP) with limited avian virulence potential in AMCRs[41]. The attenuated phenotype was subsequently attributed to both the NYP mutation in addition to a prM mutation and full attenuation effects were observed in the presence of both mutations in AMCRs, HOSPs and House finches[41]. Significantly lower viral loads in young chicks inoculated with a non-glycosylated (NYP) WNV plaque variant when compared to glycosylated (NYS) WNV have also been reported[19] leading the investigators to ascribe the phenotypic differences to the loss of glycosylation; however, other sequence alterations in the plaque variants and the specific effect of the amino acid polymorphism, independent of glycosylation, could mediate these phenotypes. Herein, the most significantly debilitated phenotypes in AMCRs were observed in birds inoculated with the KYS and NYP mutants that both lacked glycosylation signals but were imparted by different mutations within the glycosylation signal sequence. Alternative mutations that also ablated glycosylation by different amino acid substitutions at these same loci, SYS/NYF, had minor effects on avian viremia potential in AMCRs. Similar observations were made with HOSPs with the exception of the SYS mutant which showed an attenuated viremia much like KYS and NYP.As potential further support of the role of the amino acid identity at these positions for dictating phenotypes independent of glycosylation status, it is intriguing that both mutants that showed the most significant mosquito and avian competence differences KYS and NYP also encoded the most non-conservative amino acid substitutions. These consisted of a polar, uncharged asparagine substituted with a positively charged lysine (E-N154K) or a polar uncharged serine substituted with an aromatic ringed, hydrophobic proline (E-S156P) for KYS and NYP mutants, respectively.

The specific amino acid identity at the E-156 locus influenced mosquito oral infectivity independent of glycosylation status as the NYT virus had a higher infection rate than NYS. The NYP mutant exhibited a severely debilitated infection and dissemination phenotype while the NYF did not significantly affect these phenotypes compared to the NYS virus. The E-154 locus showed similar variable effects on vector competence with restricted infectivity of the KYS mutant observed when compared to the SYS mutant. With the exception of the NYP mutant virus, absence of E protein glycosylation did not significantly attenuate viral growth, infection and/or dissemination rates of NYF, SYS, SYP and KYS viruses when compared with NYS parental WNV in *Cx*. *quinquefasciatus*. Even after bypassing the midgut barrier of infection by intrathoracic inoculation, NYP produced significantly lower viral titers in the saliva. The attenuated profile of NYP virus in *Culex* spp. in the present study could explain the low frequency of isolation of WNV-NYP in nature[19, 21]. While NYP avian serum titers could presumably overcome WNV oral infection thresholds[42] in highly competent avian hosts[21], AMCRs elicited lower serum viremias with this virus. Furthermore, the restricted dissemination and transmission in alternative mosquito vectors such as *Cx*. *tarsalis* could preclude subsequent transmission. In contrast to our study, Murata et al reported the isolation and characterization of a small plaque variant with the NYP motif where no oral infectivity differences were observed with *Culex pipiens pallens* and disseminated viral titers were indistinguishable between the NYS and NYP viruses[19]. Alternative mosquito species used and viral genetic differences between the NYP viruses used in the two studies could explain the difference in results. Of note, the SYP virus that was generated as a double mutant to prevent reversion to glycosylated motif as well as to assess the duplicative effect of altering more than one amino acid within the motif did not exhibit an attenuated mosquito infection phenotype as observed for NYP. The additional E-154 mutation could have served to compensate for negative fitness effects of the E-S156P substitution possibly through structural modulation of the envelope surface projections. The lack of significant differences between NYS and NYF, SYS, SYP and KYS viruses suggests that WNV N-linked glycosylation is not a critical determinant of vector competence. The WN02 genotype that displaced the introduced WNV-NY99 genotype, is characterized principally by the incorporation of a nonsynonymous valine to alanine substitution at position E-159[11, 43]. This new genotype has been reported to exhibit a shorter extrinsic incubation period in certain *Culex* spp. and more efficient transmission rates at warmer temperatures[44]. This E-159 locus, similar to the E-154-156 motif, is present on the surface of the mature envelope protein and could indicate that numerous amino acid variants in this surface exposed envelope domain could significantly modulate vector competence phenotypes. Taken together, the data indicated that specific amino acid identities at E154 or E-156 and potential other sites on the WNV E protein could be critical for modulation of mosquito oral infectivity and vector competence exclusive of the glycosylation status of the E protein.

Interestingly, the midgut infection rate for NYT (70%) was more than twice that of the NYS virus (34%), indicating that a single amino acid polymorphism at E-156 that maintained glycosylation status of the E protein could result in a significant effect on vector competence. Nevertheless, the role of glycosylation for modulation of this phenotype between these viruses cannot be discounted as *in vitro* studies with recombinant rabies virus glycoproteins have shown that differential glycosylation efficiencies of the N-linked moieties were dependent upon the hydroxyl amino acid in the glycosylation motif[45]. That study also showed that NYT motifs were preferentially and more efficiently glycosylated than the NYS motif[45, 46]. Thus it is possible that higher glycosylation efficiency of NYT versus NYS potentially enhanced virus entry, release and/or subsequent infection rates in mosquitoes in the present study[7]. That being said, while the infection rate for NYT was significantly higher than NYS, NYT viral titers, dissemination and transmission rates remained similar to NYS. While no naturally occurring NYT WNV variants have been described, other flaviviruses such as Zika (NDT), dengue (NET), Wesselbron (NHT), Sepik (NHT), Rocio (NYT), Spondweni (NDT) and Ilheus (NYT) viruses have been shown to encode a threonine at the hydroxyl amino acid of the N-linked glycosylation motif[24].

Other flaviviruses such as Yellow fever virus and St. Louis encephalitis viruses do not encode a glycosylated E protein[10, 47, 48]. Furthermore, non-glycosylated SLEV maintains a lower oral infection threshold, i.e. has been shown to be more infectious, in *Culex* mosquitoes when compared with WNV[42], again demonstrating that the N-linked glycosylation is not a requisite for flavivirus infection of and transmission by mosquitoes. Reports by others have also demonstrated variable vector competence rates with non-glycosylated WNV isolates[49, 50] and in this study, with the exception of NYP and A’DEL all other non-glycosylated viruses were transmitted by *Cx quinquefasciatus*. The effect of the different E154-156 mutations on avian viremia potential was variable and species-specific, indicating that different mutations could be competent for avian viremia generation in certain avian species. Despite having delayed viremia onset, even the A’DEL mutant was capable of eliciting avian viremia conducive for mosquito infection, although this mutant failed to demonstrate transmissibility in *Cx quinquefasciatus*. Data presented herein indicates that WNV E154-156 motif amino identity, rather than specific N-linked glycosylation, dictates acute viral titers in birds and vector competence in mosquitoes and the effect of a particular E154-156 moiety expressed on WNV avian and vector competence was species-dependent. The isolation of many of these E-154-156 mutants in the field is intriguing and coupled with the findings here indicates the strong possibility that non-glycosylated WNV variants could circulate at relatively high frequency. The frequency of WNV genotypes to express a particular E-glycosylation motif (E154-E156) is likely due to selection pressures on viral replicative fitness by the dominant amplification host(s) and vectors utilized during local transmission events. Future studies should be designed to assess the potential fitness advantages for these variants in specific enzootic vectors and/or hosts.

## References

[1] Smithburn KC, Hughes TP, Burke AW, Paul JH. A Neurotropic Virus Isolated from the Blood of a Native of Uganda. Am J Trop Med. 1940;s1-20(4):471–92.

[2] Hubalek Z, Halouzka J. West Nile fever—a reemerging mosquito-borne viral disease in Europe. Emerg Infect Dis. 1999;5(5):643–50. 10.3201/eid0505.990505 10511520 PMC2627720

[3] Marfin AA, Petersen LR, Eidson M, Miller J, Hadler J, Farello C, et al Widespread West Nile virus activity, eastern United States, 2000. Emerg Infect Dis. 2001;7(4):730–5. 10.3201/eid0704.010423 . 11585539 PMC2631748

[4] Hayes C. West Nile Fever The Arboviruses: Epidemiology and Ecology. 1 Boca Raton: CRC Press; 1988 p. 59–88.

[5] Lindenbach B, Thiel H, Rice C. Flaviviridae: the viruses and their replication. Fields Virology. 2007:1101–52. Epub 5.

[6] Brett D. Lindenbach H-JT, Charles M. Rice. Section II: Flaviviridae In: David M. Knipe PMH, editor. Fields Virology. 1: Lippincott Williams & Wilkins.

[7] Hanna SL, Pierson TC, Sanchez MD, Ahmed AA, Murtadha MM, Doms RW. N-Linked Glycosylation of West Nile Virus Envelope Proteins Influences Particle Assembly and Infectivity. Journal of Virology. 2005;79(21):13262–74. 10.1128/JVI.79.21.13262-13274.2005 16227249 PMC1262570

[8] Mukhopadhyay S, Kim B-S, Chipman PR, Rossmann MG, Kuhn RJ. Structure of West Nile Virus. Science. 2003;302(5643):248–. 10.1126/science.1089316 14551429

[9] Berthet FX, Zeller HG, Drouet MT, Rauzier J, Digoutte JP, Deubel V. Extensive nucleotide changes and deletions within the envelope glycoprotein gene of Euro-African West Nile viruses. Journal of General Virology. 1997;78(9):2293–7. 10.1099/0022-1317-78-9-2293 9292017

[10] Adams SC, Broom AK, Sammels LM, Hartnett AC, Howard MJ, Coelen RJ, et al Glycosylation and antigenic variation among Kunjin virus isolates. Virology. 1995;206(1):49–56. Epub 1995/01/10. doi: S0042-6822(95)80018-2 [pii]. . 7530394 10.1016/s0042-6822(95)80018-2

[11] Ebel G, Carricaburu J, Young D, Bernard K, Kramer L. Genetic and phenotypic variation of West Nile virus in New York, 2000–2003. Am J Trop Med Hyg. 2004;71(4):493–500. 15516648

[12] Lanciotti R, Roehrig J, Deubel V, Smith J, Parker M, Steele K, et al Origin of the West Nile virus responsible for an outbreak of encephalitis in the northeastern United States. Science. 1999;286(5448):2333–7. 10.1126/science.286.5448.2333 10600742

[13] Bin H, Grossman Z, Pokamunski S, Malkinson M, Weiss L, Duvdevani P, et al West Nile fever in Israel 1999–2000: from geese to humans. Ann N Y Acad Sci. 2001;951:127–42. 10.1111/j.1749-6632.2001.tb02691.x 11797770

[14] Murgue B, Murri S, Triki H, Deubel V, Zeller HG. West Nile in the Mediterranean Basin: 1950–2000. Annals of the New York Academy of Sciences. 2001;951(1):117–26. 10.1111/j.1749-6632.2001.tb02690.x 11797769

[15] Beasley DWC, Whiteman MC, Zhang S, Huang CYH, Schneider BS, Smith DR, et al Envelope Protein Glycosylation Status Influences Mouse Neuroinvasion Phenotype of Genetic Lineage 1 West Nile Virus Strains. J Virol. 2005;79(13):8339–47. 10.1128/JVI.79.13.8339-8347.2005 15956579 PMC1143769

[16] Shirato K, Miyoshi H, Goto A, Ako Y, Ueki T, Kariwa H, et al Viral envelope protein glycosylation is a molecular determinant of the neuroinvasiveness of the New York strain of West Nile virus. J Gen Virol. 2004;85(12):3637–45. 10.1099/vir.0.80247-0 15557236

[17] Beasley D, Davis C, Estrada-Franco J, Navarro-Lopez R, Campomanes-Cortes A, Tesh R, et al Genome sequence and attenuating mutations in West Nile virus isolate from Mexico. Emerg Infect Dis. 2004;10(12):2221–4. 10.3201/eid1012.040647 15663867 PMC3323401

[18] Alsaleh K, Khou C, Frenkiel M-P, Lecollinet S, Vàzquez A, de Arellano ER, et al The E glycoprotein plays an essential role in the high pathogenicity of European–Mediterranean IS98 strain of West Nile virus. Virology. 2016;492(Supplement C):53–65. 10.1016/j.virol.2016.02.009. 26896935

[19] Murata R, Eshita Y, Maeda A, Maeda J, Akita S, Tanaka T, et al Glycosylation of the West Nile Virus Envelope Protein Increases In Vivo and In Vitro Viral Multiplication in Birds. Am J Trop Med Hyg. 2010;82(4):696–704. 10.4269/ajtmh.2010.09-0262 20348522 PMC2844573

[20] Kariwa H, Murata R, Totani M, Yoshii K, Takashima I. Increased Pathogenicity of West Nile Virus (WNV) by Glycosylation of Envelope Protein and Seroprevalence of WNV in Wild Birds in Far Eastern Russia. International Journal of Environmental Research and Public Health. 2013;10(12):7144 10.3390/ijerph10127144 24351738 PMC3881158

[21] Langevin S, Bowen R, Ramey W, Sanders T, Maharaj P, Fang Y, et al Envelope and pre-membrane structural amino acid mutations mediate diminished avian growth and virulence of a Mexican West Nile virus isolate. Journal of General Virology. 2011 10.1099/vir.0.035535-0 PMC3352571 21865445

[22] Moudy RM, Zhang B, Shi P-Y, Kramer LD. West Nile virus envelope protein glycosylation is required for efficient viral transmission by Culex vectors. Virology. 2009;387(1):222–8. 10.1016/j.virol.2009.01.038 19249803 PMC2742948

[23] Moudy RM, Payne AF, Dodson BL, Kramer LD. Requirement of Glycosylation of West Nile Virus Envelope Protein for Infection of, but Not Spread within, Culex quinquefasciatus Mosquito Vectors. The American journal of tropical medicine and hygiene. 2011;85(2):374–8. 10.4269/ajtmh.2011.10-0697 21813861 PMC3144839

[24] Wen D, Li S, Dong F, Zhang Y, Lin Y, Wang J, et al N-glycosylation of Viral E Protein Is the Determinant for Vector Midgut Invasion by Flaviviruses. mBio. 2018;9(1). 10.1128/mBio.00046-18 29463651 PMC5821097

[25] Fontes-Garfias CR, Shan C, Luo H, Muruato AE, Medeiros DBA, Mays E, et al Functional Analysis of Glycosylation of Zika Virus Envelope Protein. Cell Reports. 2017;21(5):1180–90. 10.1016/j.celrep.2017.10.016 29091758 PMC5708593

[26] Lanciotti RS, Ebel GD, Deubel V, Kerst AJ, Murri S, Meyer R, et al Complete Genome Sequences and Phylogenetic Analysis of West Nile Virus Strains Isolated from the United States, Europe, and the Middle East. Virology. 2002;298(1):96–105. 10.1006/viro.2002.1449. 12093177

[27] Scherret JH, Mackenzie JS, Khromykh AA, Hall RA. Biological Significance of Glycosylation of the Envelope Protein of Kunjin Virus. Annals of the New York Academy of Sciences. 2001;951(1):361–3. 10.1111/j.1749-6632.2001.tb02719.x 11797800

[28] Kinney RM, Huang CY, Whiteman MC, Bowen RA, Langevin SA, Miller BR, et al Avian virulence and thermostable replication of the North American strain of West Nile virus. J Gen Virol. 2006;87(Pt 12):3611–22. 10.1099/vir.0.82299-0 . 17098976

[29] Brault AC, Langevin SA, Bowen RA, Panella NA, Biggerstaff BJ, Miller BR, et al Differential virulence of West Nile strains for American crows. Emerg Infect Dis. 2004;10(12):2161–8. Epub 2005/01/25. 10.3201/eid1012.040486 15663854 PMC1237116

[30] Sbrana E, Tonry JH, Xiao S-Y, Da Rosa APAT, Higgs S, Tesh RB. Oral transmission of West Nile virus in a hamster model. Am J Trop Med Hyg. 2005;72(3):325–9. 15772330

[31] Bellamy R, Kardos E. A strain of *Culex tarsalis* Coq. reproducing without blood meals. Mosquito News. 1958;18(2):132–5.

[32] Maharaj PD, Bolling BG, Anishchenko M, Reisen WK, Brault AC. Genetic determinants of differential oral infection phenotypes of West Nile and St. Louis encephalitis viruses in Culex spp. mosquitoes. Am J Trop Med Hyg. 2014;91(5):1066–72. 10.4269/ajtmh.14-0289 25157120 PMC4228875

[33] Frost MJ, Zhang J, Edmonds JH, Prow NA, Gu X, Davis R, et al Characterization of Virulent West Nile Virus Kunjin Strain, Australia, 2011. Emerging Infectious Diseases. 2012;18(5):792–800. 10.3201/eid1805.111720 PMC3358055. 22516173 PMC3358055

[34] Beasley DW, Whiteman MC, Zhang S, Huang CY, Schneider BS, Smith DR, et al Envelope protein glycosylation status influences mouse neuroinvasion phenotype of genetic lineage 1 west nile virus strains. J Virol. 2005;79(13):8339–47. 10.1128/JVI.79.13.8339-8347.2005 . 15956579 PMC1143769

[35] Dietrich EA, Bowen RA, Brault AC. An ex vivo avian leukocyte culture model for West Nile virus infection. J Virol Methods. 2015;218:19–22. 10.1016/j.jviromet.2015.03.004 . 25783683 PMC4583197

[36] Dietrich EA, Langevin SA, Huang CY, Maharaj PD, Delorey MJ, Bowen RA, et al West Nile Virus Temperature Sensitivity and Avian Virulence Are Modulated by NS1-2B Polymorphisms. PLoS Negl Trop Dis. 2016;10(8):e0004938 Epub 2016/08/23. 10.1371/journal.pntd.0004938 27548738 PMC4993437

[37] Langevin SA, Bowen RA, Reisen WK, Andrade CC, Ramey WN, Maharaj PD, et al Host competence and helicase activity differences exhibited by West Nile viral variants expressing NS3-249 amino acid polymorphisms. PLoS One. 2014;9(6):e100802 10.1371/journal.pone.0100802 24971589 PMC4074097

[38] Komar N, Langevin S, Hinten S, Nemeth N, Edwards E, Hettler D, et al Experimental infection of North American birds with the New York 1999 strain of West Nile virus. Emerg Infect Dis. 2003;9(3):311–22. 10.3201/eid0903.020628 . 12643825 PMC2958552

[39] Li J, Bhuvanakantham R, Howe J, Ng M-L. The glycosylation site in the envelope protein of West Nile virus (Sarafend) plays an important role in replication and maturation processes. Journal of General Virology. 2006;87(3):613–22. 10.1099/vir.0.81320-0 16476982

[40] Andrade CC, Maharaj PD, Reisen WK, Brault AC. North American West Nile virus genotype isolates demonstrate differential replicative capacities in response to temperature. Journal of General Virology. 2011;92(11):2523–33. 10.1099/vir.0.032318-0 21775581 PMC3352365

[41] Langevin SA, Bowen RA, Ramey WN, Sanders TA, Maharaj PD, Fang Y, et al Envelope and pre-membrane protein structural amino acid mutations mediate diminished avian growth and virulence of a Mexican West Nile virus isolate. Journal of General Virology. 2011;92(12):2810–20. 10.1099/vir.0.035535-0 21865445 PMC3352571

[42] Maharaj PD, Bolling BG, Anishchenko M, Reisen WK, Brault AC. Genetic Determinants of Differential Oral Infection Phenotypes of West Nile and St. Louis Encephalitis Viruses in Culex spp. Mosquitoes. The American Journal of Tropical Medicine and Hygiene. 2014;91(5):1066–72. 10.4269/ajtmh.14-0289 25157120 PMC4228875

[43] Davis CT, Ebel GD, Lanciotti RS, Brault AC, Guzman H, Siirin M, et al Phylogenetic analysis of North American West Nile virus isolates, 2001–2004: Evidence for the emergence of a dominant genotype. Virology. 2005;342(2):252–65. 10.1016/j.virol.2005.07.022 16137736

[44] Kilpatrick AM, Meola MA, Moudy RM, Kramer LD. Temperature, Viral Genetics, and the Transmission of West Nile Virus by Culex pipiens Mosquitoes. PLOS Pathogens. 2008;4(6):e1000092 10.1371/journal.ppat.1000092 18584026 PMC2430533

[45] Kasturi L, Eshleman JR, Wunner WH, Shakin-Eshleman SH. The Hydroxy Amino Acid in an Asn-X-Ser/Thr Sequon Can Influence N-Linked Core Glycosylation Efficiency and the Level of Expression of a Cell Surface Glycoprotein. Journal of Biological Chemistry. 1995;270(24):14756–61. 10.1074/jbc.270.24.14756 7782341

[46] Shakin-Eshleman SH, Spitalnik SL, Kasturi L. The Amino Acid at the X Position of an Asn-X-Ser Sequon Is an Important Determinant of N-Linked Core-glycosylation Efficiency. Journal of Biological Chemistry. 1996;271(11):6363–6. 10.1074/jbc.271.11.6363 8626433

[47] Mossenta M, Marchese S, Poggianella M, Slon Campos JL, Burrone OR. Role of N-glycosylation on Zika virus E protein secretion, viral assembly and infectivity. Biochemical and Biophysical Research Communications. 2017;492(4):579–86. 10.1016/j.bbrc.2017.01.022 28069378

[48] Vorndam V, Mathews JH, Barrett ADT, Roehrig JT, Trent DW. Molecular and biological characterization of a non-glycosylated isolate of St Louis encephalitis virus. J Gen Virol. 1993;74(12):2653–60. 10.1099/0022-1317-74-12-2653 7506301

[49] Fall G, Diallo M, Loucoubar C, Faye O, Sall AA. Vector Competence of Culex neavei and Culex quinquefasciatus (Diptera: Culicidae) from Senegal for Lineages 1, 2, Koutango and a Putative New Lineage of West Nile virus. The American Journal of Tropical Medicine and Hygiene. 2014;90(4):747–54. 10.4269/ajtmh.13-0405 PMC3973524. 24567319 PMC3973524

[50] Melian EB, Hall-Mendelin S, Du F, Owens N, Bosco-Lauth AM, Nagasaki T, et al Programmed Ribosomal Frameshift Alters Expression of West Nile Virus Genes and Facilitates Virus Replication in Birds and Mosquitoes. PLoS Pathog. 2014;10(11):e1004447 10.1371/journal.ppat.1004447 25375107 PMC4223154

